# Higher social tolerance in wild versus captive common marmosets: the role of interdependence

**DOI:** 10.1038/s41598-020-80632-3

**Published:** 2021-01-12

**Authors:** Francisco Edvaldo de Oliveira Terceiro, Maria de Fátima Arruda, Carel P. van Schaik, Arrilton Araújo, Judith Maria Burkart

**Affiliations:** 1 Department of Physiology and Behaviour, Universidade Federal do Rio Grande do Norte, Av. Sen. Salgado Filho, 3000 - Candelária, Natal, RN 59064-741 Brazil; 2 Department of Anthropology, Universität Zürich, Winterthurerstrasse 190, 8057 Zürich, Switzerland

**Keywords:** Anthropology, Animal behaviour

## Abstract

Social tolerance in a group reflects the balance between within-group competition and interdependence: whereas increased competition leads to a reduction in social tolerance, increased interdependence increases it. Captivity reduces both feeding competition and interdependence and can therefore affect social tolerance. In independently breeding primates, social tolerance has been shown to be higher in captivity, indicating a strong effect of food abundance. It is not known, however, how social tolerance in cooperative breeders, with their much higher interdependence, responds to captivity. Here, we therefore compared social tolerance between free-ranging and captive groups in the cooperatively breeding common marmoset and found higher social tolerance (measured as proximity near food, co-feeding, and food sharing) in the wild. Most likely, social tolerance in the wild is higher because interdependence is particularly high in the wild, especially because infant care is more costly there than in captivity. These results indicate that the high social tolerance of these cooperative breeders in captivity is not an artefact, and that captive data may even have underestimated it. They may also imply that the cooperative breeding and foraging of our hominin ancestors, which relied on strong interdependence at multiple levels, was associated with high social tolerance.

## Introduction

Living in a stable social group requires a minimum of social tolerance^1^. The degree of social tolerance in a group reflects the balance between within-group competition over resources, and the need for group members as cooperation partners, generally to cooperate in between-group encounters, or to avoid predators^2^. Both factors can vary within species, and the phenotypic responses to such variation can inform us about their relative importance. Substantial variation is manifest in the contrast between wild and captive conditions: In captivity, feeding competition is reduced because of systematic provisioning by humans. This generally should lower tension between individuals, particularly around food, and thus increase social tolerance. Interdependence is reduced as well in captivity: there is no need to cooperate with group members in between-group conflicts or to avoid predators. In addition, interdependence is reduced even more in captivity in species with shared infant care, because infants don’t have to be carried over long distances, they don’t have to be protected from predation, and provisioning them with food is less costly because the food is readily made available by human caretakers. Thus, the influence of reduced competition in captivity will increase social tolerance, whereas the influence of reduced interdependence will decrease it.

Social tolerance can readily be measured in foraging contexts focusing on co-feeding, food sharing, and proximity in food patches^3–6^. In independently breeding red-fronted and ring-tailed lemurs^7^, a direct comparison revealed higher levels of social tolerance in captive compared to free-ranging groups. Likewise, in orangutans, active food sharing can be common in captivity^8^ but is quite rare in the wild^9^, and the same pattern has also been reported for chimpanzees^10^. These results suggest that in these independently breeding primates, the effect on social tolerance of reduced feeding competition outweighs the effect of reduced interdependence.

The life of cooperatively breeding callitrichid monkeys, which include marmosets and tamarins, is marked by high levels of social tolerance and even prosociality^6,11–14^ even though on rare occasions it is interrupted by fierce competition for breeding positions, in particular in females^15–20^. The peacefulness of cooperative breeders^13^ during everyday life appears to be important in facilitating several activities that demand fine-tuned coordination and cooperation. For instance, infant carrying is crucial for infant survival and shared among all group members. It can only be performed properly if handovers from one carrier to the next happen without lengthy and/or noisy interruptions, which requires social tolerance and behavioural coordination in proximity among all group members and close social monitoring in non-stressful situations^21^. Accordingly, across primates, cooperative breeding is positively correlated with social tolerance^11,22^. This finding is intriguing because it can contribute to understanding the evolution of the high levels of social tolerance in humans who also engage in cooperative breeding^23,24^. However, this comparative data was all collected in captive populations, which is problematic if different species react differently to captivity, because the balance of within-group competition and interdependence in the wild versus captivity changes differently.

Our goal was therefore to compare social tolerance in captive versus free-ranging groups of common marmosets. They live in various habitats in north-eastern Brazil, in small groups of 5–17 individuals^25,26^, typically composed of a single breeding pair as well as immatures and sexually mature helpers of both sexes^15,17,25,27^. Helpers are predominantly adult offspring from the breeding pair but also occasionally immigrants from other groups^28^. Based on the results with the independently breeding lemurs, orangutans, and chimpanzees, we expected higher tolerance in captivity. However, since as cooperative breeders, interdependence is particularly high in callitrichids, we anticipated this effect to be lower, and perhaps outweighed by the reduced interdependence in captivity.

Interdependence (i.e. that an individual’s fitness is dependent on another’s fitness^29,30^) is clearly high in cooperatively breeding species, since help in infant care by non-breeders strongly affects group members’ inclusive fitness^31^. Different cooperatively breeding callitrichid monkey species vary in the dependence of mothers on others to rear their offspring and therefore also the extent to which group members’ inclusive fitness is interdependent. A recent study found that higher levels of interdependence among callitrichid species was indeed linked to higher tolerance among adults (adult-adult food sharing^32^). This important role of interdependence therefore suggests that its reduction in captivity may even lead to lower social tolerance.

To compare free-ranging and captive marmosets, we adapted the arena approach developed to compare social tolerance in free-ranging and captive lemurs^7^, which has been successfully validated with other quantitative measures of social tolerance^3,4^. We repeatedly presented multiple groups in the wild and in captivity with 1m^2^ arenas baited with pieces of banana (the same number as non-infant individuals in the group) and subsequently coded the marmosets’ behaviour from video recordings. Social tolerance is operationalized as spending more time on the arena, individuals having more equal access to the resource^4,11^, more co-feeding and food sharing, and less frequent aggression and evasion (e.g. grabbing a piece of banana and running away with it).

## Results

### Principal component analysis

We analysed 133 arena sessions (80 in 4 captive groups and 53 in 2 free-ranging groups). We first performed a Principal Component Analysis of the separate behaviours. Parallel analyses (see supplementary material, supplementary Figure 1) yielded two PCA components (Table 1). The first component (explaining 30.6% of variance) had high positive loadings on time spent on the arena and negative loadings on evasive behaviours and was thus named ***spatial tolerance*** (see also supplementary Table 1). The second component (explaining 25.8% of variance) loaded highly on food sharing and co-feeding and was named ***feeding tolerance*** (see also supplementary Table 2).

**Figure 1:**
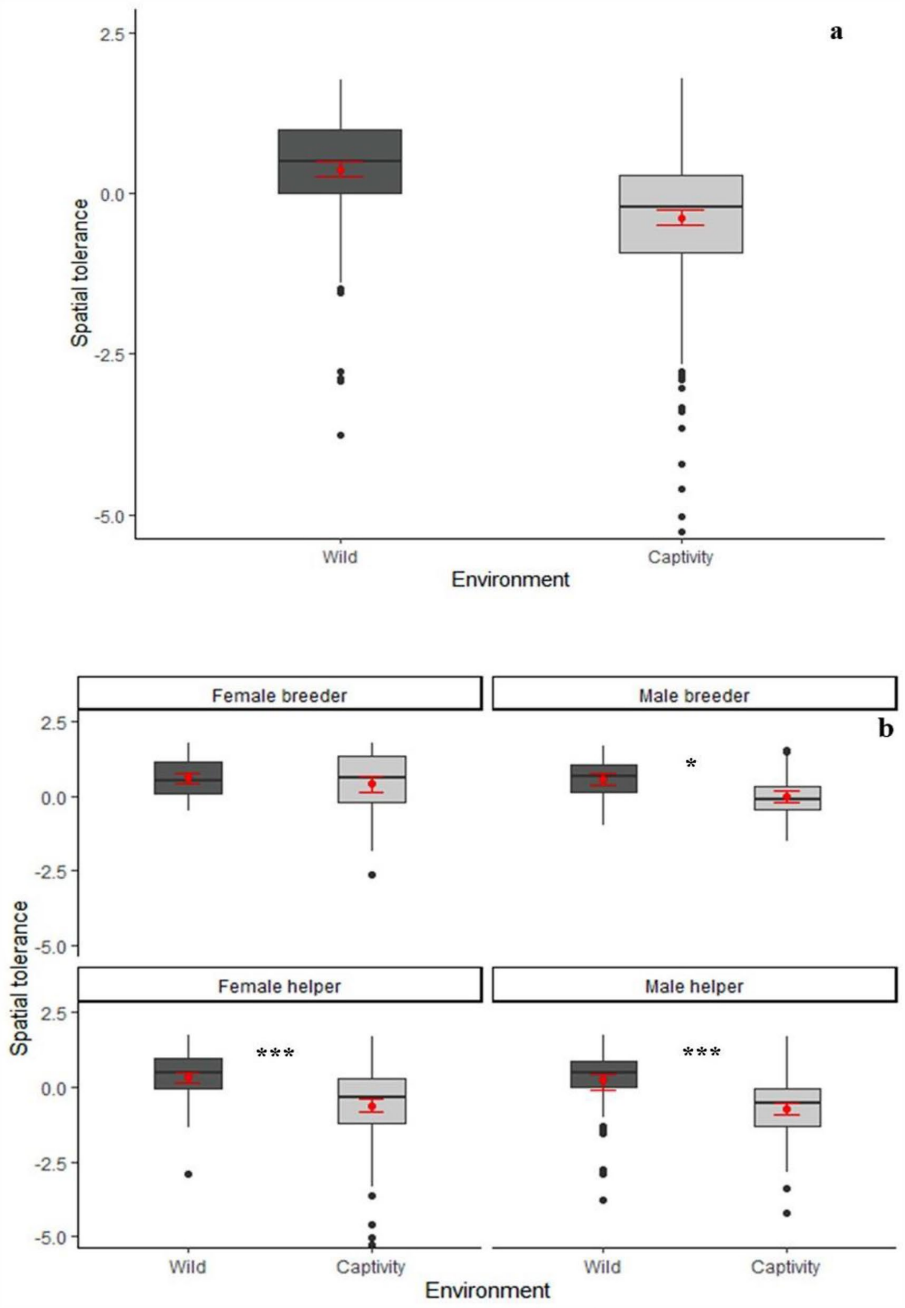
*Spatial tolerance* (PC1), split up according to (**a**) environment and (**b**) sex-status categories. *p < 0.05, **p < 0.01, ***p < 0.001, (Games-Howell post-hoc tests). Black lines indicate medians; upper and lower edges of boxes indicate upper and lower quartiles; whiskers indicate the ranges for the bottom and top 25% of the data values, excluding outliers; black dots indicate outliers; red dots indicate mean value and red bars indicate SEM (N = 46). Software R Studio version 1.1.463. https://www.r-project.org/.

**Table 1:** Principal component analysis.

Principal Component Analysis	PC1 “Spatial tolerance”	PC2 “Feeding tolerance”
*Time spent on arena*	**0.580**	0.307
*Evasive behaviours*	− **0.630**	− 0.018
*Food sharing*	− 0.413	**0.568**
*Co-feeding*	− 0.210	**0.701**
*Agonism*	− 0.235	− 0.146

The entries of the behaviours (italics) are loadings, with those above 0.5 highlighted in bold.

**Table 2:** Anova table on model for ***spatial tolerance (PC1) ***and ***feeding tolerance (PC2).***

Fixed factor	NumDF	DenDF	F-value	*p* value
**Model PC 1 (Spatial tolerance)**				
Environment	1	3.6323	2.237	0.216
*Sex-Status*	3	27.2186	8.923	0.0003***
Environment*Sex-Status	3	27.2186	3.1612	0.040*
**Model PC 2 (Feeding tolerance)**				
Environment	1	12.06	0.493	0.50
Status	1	742.83	1.594	0.21
Environment*Status	1	742.83	17.505	3.208e−05***

Bold values indicate *p* < 0.05.

### Spatial and feeding tolerance

To explain variation in ***spatial tolerance,*** we selected the best fitting linear mixed model including the following variables: *environment* (wild/captivity), *sex-status* (female breeder/male breeder/female helper/male helper), *presence of infants in the group* (yes/no), and *sex ratio* (number of adult males : adult females). The best-fitting model explained the data better than the null model (X^2^ (7) = 24.14, *p* < 0.0001, Table 2) and included *environment*, *sex-status* and their interaction. As shown in Fig. 1, animals in the wild spent more time on the arena overall, except for breeding females (Games-Howell post hoc test, Supplementary Table 1). These results were corroborated by separate analyses of the raw variables *time spent on arena* and *evasive behaviours* (Supplementary Tables 3–6; supplementary Figures 2 and 3).

**Table 3:** Fligner–Killeen test for homogeneity of variances.

Comparison	Chi-squared	df	*p* value
**Fligner-Killeen Test for homogeneity of variances**			
All Wild versus Captivity	24.70	1	**6.71e−07**
Wild FB versus MB versus FH versus MH	1.27	3	0.73
Captivity FB versus MB versus FH versus MH	14.17	3	**0.0027**

**Figure 2:**
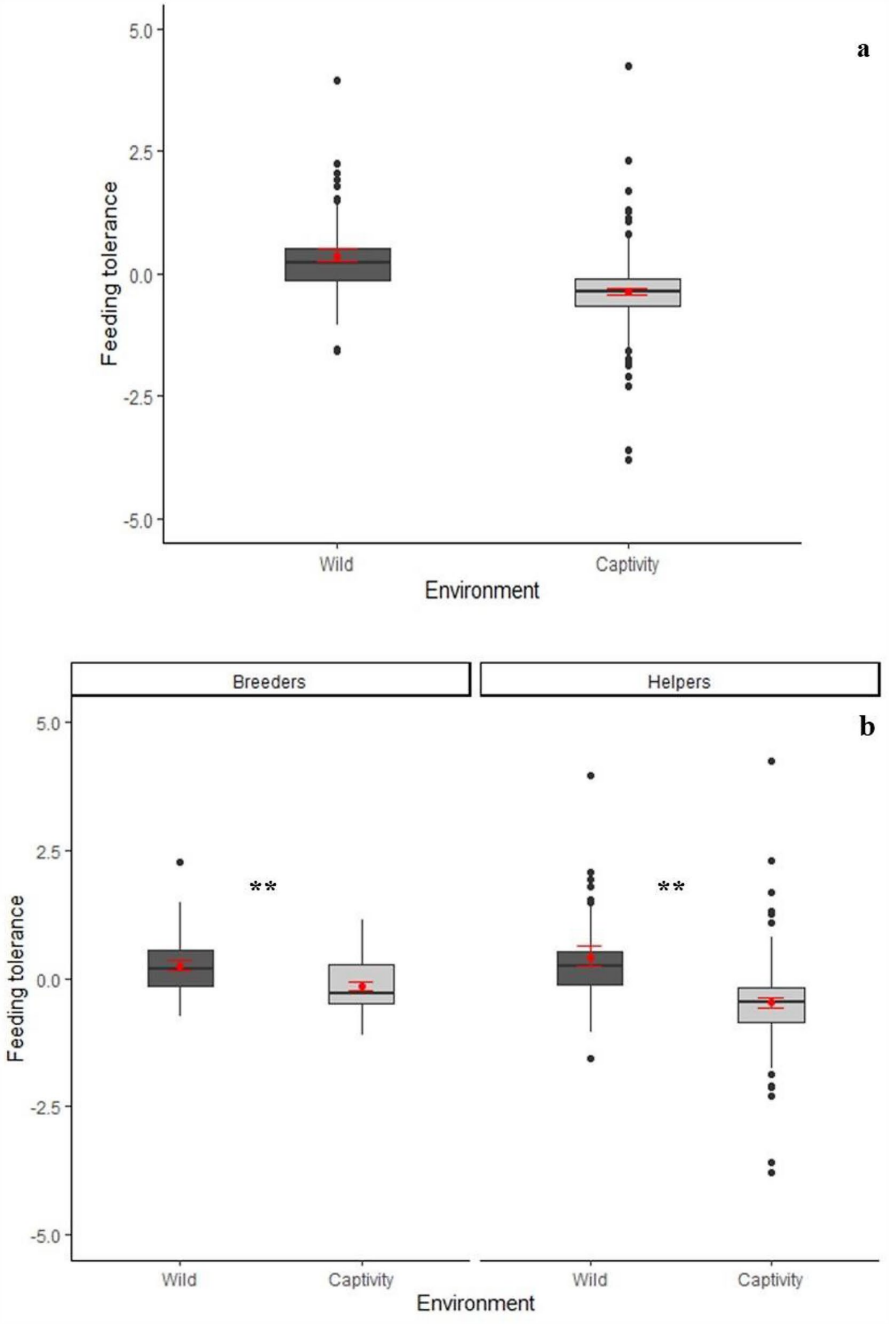
*Feeding tolerance* (PC2), split up according to (**a**) environment and (**b**) status categories. *p < 0.05, **p < 0.01, ***p < 0.001, (Games-Howell post-hoc tests). Black lines indicate medians; upper and lower edges of boxes indicate upper and lower quartiles; whiskers indicate the ranges for the bottom and top 25% of the data values, excluding outliers; black dots indicate outliers; red dots indicate mean value and red bars indicate SEM (N = 46). Software R Studio version 1.1.463. https://www.r-project.org/.

To further examine the difference in ***spatial tolerance*** between captive and free-ranging groups, we also tested whether group members in the wild were not only more, but also more equally present on the arena (Fig. 1b; see also supplementary Figure 2). A Fligner-Killeen test (Table 3) revealed different variance distributions in the wild versus captivity, with lower levels of variance in the wild (wild: 0.91; captivity: 1.66). Next, we asked whether this wild-captive difference also held at the level of sex-status categories. A Fligner-Killeen test showed that the variances across the four categories were not different in the wild but were different in captivity (Table 3), where helpers had more variable access to the arena than breeders.

To explain variation in ***feeding tolerance,*** we selected the best-fitting linear mixed model including the same variables as above. This model explained the data better than the null model (X^2^ (6) = 25.842, *p* < 0.0001, Table 2) and included *environment*, *status* (breeders/helpers) and the interaction between these two. As shown in Fig. 2, ***feeding tolerance*** was higher in the wild than in captivity, in both breeders and helpers (Games-Howell post-hoc, supplementary Table 2). Again, we corroborated these results by separate analyses of the raw variables. The variable *co-feeding* showed a highly comparable pattern.

*Agonistic behaviours*, which loaded weakly negative on both PC1 (***Spatial tolerance***) and PC2 *(feeding tolerance), *were very rare overall and did not differ between the environments (Supplementary Tables 7–10; supplementary Figures 4 and 5).

These results strongly suggest that social tolerance was higher in the wild compared to captivity. Alternatively, however, they could be an artefact of variation in participation in the experiment (i.e. visiting the arena at all during a given session), the presence of infants in the group, or sex ratio, which arguably could influence social tolerance^15,33^. We therefore next examined these alternatives. First, because not all individuals participated in every experimental session (Supplementary Table 11), we performed a binomial GLMM to test whether participation in the experiment differed between the wild and captivity. This was not the case, although participation was slightly, non-significantly higher in captivity. Notably, if this difference had influenced the main results, it would have biased the results towards higher ***spatial tolerance*** in captivity compared to the wild, whereas in fact we found the opposite (Fig. 1a). Second, groups that had infants during experiments had a lower participation than those that did not have them. This pattern might have arisen most likely because individuals carrying infants avoided coming down to the food arenas. Finally, sex ratio had a negative effect on arena presence (Supplementary Table 12 and supplementary Figure 6). However, neither variable was included into the best models for ***spatial tolerance*** and ***feeding tolerance*** (Table 2), confirming that our results are robust.

## Discussion

We compared social tolerance in a cooperative breeder, the common marmoset in wild versus captive conditions with a foraging arena approach^7^. We found two dimensions of social tolerance: spatial tolerance and feeding tolerance. In contrast to independently breeding primates who were more tolerant in captivity, the cooperatively breeding marmosets scored higher on both dimensions in the wild. First, with regard to spatial tolerance, free-ranging marmosets, in particular male breeders and helpers of either sex, spent more time on the feeding arena (Fig. 1a,b), and their presence there was also more evenly distributed among individuals than in captivity (Table 3). Second, with regard to feeding tolerance, all adults, both breeders and helpers, scored higher in the wild than their captive counterparts (Table 2 and Fig. 2b). Separate analyses of the behaviours constitutive of spatial and feeding tolerance corroborate these findings (Supplementary Tables 3–10 and supplementary Figures 2–5). Despite higher levels of social tolerance in the wild and somewhat intensified animosity among the captive marmosets, the basic patterns of sociality on both environments remained the same as expected from an ample literature on this topic^17,32,34–39^. Moreover, even though the longer presence on the arena in the wild could provide more opportunity for conflict, this was not observed. Instead, it seems that prosocial motivation remained high in both environments and was even higher for helpers in the natural environment.

These results show that captive conditions with ample provisioning from humans and no predation decreases, rather than increases social tolerance in this cooperative breeder. This result clearly differs from the pattern among independently breeding primates, who show higher tolerance in captivity^7–10^, most likely because of reduced competition over food. Marmoset social tolerance therefore is less affected by feeding competition but primarily responsive to the degree of interdependence. Interdependence is higher in the wild because infants have to be carried over longer distances, food sharing with them is more vital because of the absence of human provisioning, and group members have to collaborate more to detect and exploit food resources and to detect and mob predators^12,28,40^. Our results thus add to the evidence supporting an enhancing effect of interdependence on social tolerance in callitrichids. Furthermore, our results converge with our observations of more active food sharing among free-ranging marmosets during the harsher periods of dry season (Arruda and de Oliveira Terceiro, in prep). In particular, we find that this link not only holds inter-specifically (i.e. higher levels of adult-adult food sharing in more interdependent species:^32^), but also intra-specifically. How this flexibility is achieved remains unresolved for now. An intriguing possibility is that it is mediated through developmental effects, and that being reared as an infant in more interdependent groups leads individuals to develop into a more interdependent phenotype themselves^24,41^.

These results confirm that the high levels of social tolerance measured in callitrichids in captivity are not an artefact of extensive provisioning, and thus corroborate previous observational reports documenting high levels of social tolerance in free-ranging callitrichids^6,13^. They also corroborate broader interspecific tests based on captive data^11,33^. In fact, captive data may even underestimate the link between tolerance and cooperative breeding, because captive data appears to underestimate social tolerance in cooperative breeders but overestimate it in independent breeders. Consistent with such a pattern, the cooperatively breeding meerkats (*Suricata suricatta*) in the wild had higher grooming rates and a more cohesive social network than their captive counterparts^42^. However, future work must test the generality of the finding that captivity effects on social tolerance are positive in independent breeders^7–9,43^, but negative in cooperative breeders (this study^42^,). Such systematic tests of opposite captivity effects on social tolerance may also include lineages other than primates, e.g. canids, where social tolerance around food and prosocilaity were also higher in cooperatively breeding wolves (*Canis lupus*) than in independently breeding dogs (*Canis familiaris*;^44,45^).

Overall, our results point to a key role of mutual interdependence in the emergence of high levels of social tolerance in cooperative breeders. They therefore also have implications to understand the evolutionary origin of the high level of social tolerance in humans^46,47^. The finding that among cooperative breeders increased interdependence is associated with higher social tolerance and food sharing provides strong support for the hypothesis that cooperative breeding in our ancestors facilitated the evolution of our skill-intensive, cooperative foraging niche. This socioecological niche can yield highly varying returns, which is buffered by extensive and systematic interpersonal and intergenerational resource transfers^48^ that are only possible with very high levels of social tolerance. The hypothesis that several of the differences between humans and the other great apes can be understood as a consequence of the fact that our ancestors became cooperative breeders contributes to explaining many of our derived life history traits^49–51^ and our demographic success^49,52,53^. Moreover, it may also explain our intensive cooperation, cumulative culture and elaborate communication^23,54^. Alternatively, these behavioural differences can also be understood as the result of obligate cooperative foraging, which is unique to humans among primates^46^. These two lines of reasoning, however, have a critical commonality, namely that both pose interdependence as key factor, either reproductive or ecological interdependence. The interdependence framework^29,30^, according to which many cases of cooperative behaviours are more likely to emerge the more one individual’s fitness is dependent on another’s fitness, is thus a unifying umbrella concept that can reconcile these two perspectives.

## Methods

### Subjects

We tested a total of 48 common marmosets, 25 in the wild and 23 in captivity (Supplementary Table 11 for group composition). The free-ranging animals were tested in the Assu National Forest managed by the Chico Mendes Institute for Biodiversity (ICMBio) in the state of Rio Grande do Norte, north-eastern Brazil. This field site is a conservation unit located in the municipality of Assu, RN (5º35′ S, 36º56′ W), composed of 518 ha of arboreal shrub vegetation, typical of the Caatinga biome. This environment is characterized by high temperatures, averaging 28ºC, and low rainfall, 70 mm on average, concentrated between February and June (Instituto Nacional de Metereologia – INMET). We conducted the experiments in two free-ranging marmoset groups, a monogamous and a polygynous one. Over the period of testing, the group composition changed (Supplementary Table 11). The monogamous group ranged from five to seven individuals, and the polygynous one from 14 to 16 individuals. Both groups have been systematically followed by our team since 2017 and 2004, respectively and have varying levels of relatedness. The monogamous group has one individual unrelated to the offspring, whereas the polygynous group has several half-siblings. None of these groups was provisioned by observers, except when necessary for marking.

In captivity, we tested 23 animals in 4 groups. Unlike the free-ranging groups, all individuals are in groups with their full siblings, apart from the breeding pair. All groups were housed in home enclosures (1.8 m × 2.7 m × 2.4 m) with access to outdoor enclosures (1.8 m × 3.7 m × 2.4 m), where the experiments were performed. Home enclosures were equipped with a sleeping box, a water dispenser, several wooden climbing structures, frequently changing enrichment devices, an infrared lamp, and a mulch floor. The outdoor enclosures contained natural soil and vegetation, as well as woody climbing structures. For more details on husbandry conditions see^55^. All animals were kept in accordance with Swiss legislation. All experiments were in concordance with the ethical regulations both in Brazil from Federal University of Rio Grande do Norte ethics committee (#067.085/2017) and Switzerland from the Kantonales Veterinäramt (#183/13). Brazilian fieldwork permission from Instituto Chico Mendes de Conservação da Biodiversidade (#59,828). All methods in this manuscript were carried out in accordance with relevant guidelines and regulations cited above.

### Apparatus and procedure

To quantify social tolerance, we adapted Fichtel’s arena approach^7^. The arenas (1 × 1 m) were made of a wooden frame, with a grid attached on top of it, and were suspended and fixed with ropes approximately one meter above the ground, close to a resting tree (in the wild) or on the climbing structures of the outdoor enclosure (in captivity). The arenas were baited with as many banana pieces as non-infant individuals in each group (Supplementary Figure 7). To account for group size differences, the food pieces were distributed uniformly on the entire arena (for groups > six non-infant individuals) or on half of the arena so as to maintain the same spatial distribution of food (for groups ≤ six individuals). Each of the six groups had its own feeding arena, to avoid any scent marking from another group on the arena.

In the wild, experiments were conducted when the group was about to move to predictable destinations: when the marmosets were approaching the resting tree after morning foraging, and when the group was leaving the resting tree for afternoon foraging. The focal groups were followed before and after each trial, from waking time until sleeping time. For both groups, all possible places for resting were known from previous studies (Araujo et al*.* in prep), and the arenas were permanently installed. As soon as the group started to move towards the arena, the observer stopped following them and moved ahead to place the food pieces in anticipation of the group’s arrival. In captivity, the arenas were set up in the absence of the subjects in the outdoor enclosure, and the experiment started when the experimenter opened the door to the outdoor enclosure.

The experiments in the wild were conducted from March to August 2018 and were undertaken twice a day. The arenas were installed at the testing position and were kept there during three full days in a row. Prior to the experiments, all groups were well habituated to the arenas and the video recorder (Sony HDR-HC9E). Once habituated, we performed 30 test sessions with each wild-living group. The experiments in captivity were conducted between September and October 2018, and we performed 20 test session with each captive group. Sessions in which the entire group fled before all pieces were eaten due to external disturbance were excluded from the analysis (only in the wild: one trial in the polygynous group and six trials in the monogamous group). The experiment started when the first marmoset entered the arena and ended when all banana pieces were eaten. All experiments were video recorded, and the behaviour of all individuals quantified afterwards from the recordings. Ten percent of the trials were randomly selected and coded by two other independent coders, one for captive and another for free-ranging groups. We used Intraclass correlation coefficient (ICC) two-way random effects to assess the reliability of the ratings. The coder for captive groups reached a consistency of 99.6% on time measures and an inter-rater reliability of 83% for behaviour measures. The coder for free-ranging groups reached a consistency of 90.2% on time measurements and an inter-rater reliability of 96% for behaviour measures.

### Data coding and analysis

We used INTERACT for video analyses and defined the following behaviours:

- Time inside feeding arena: Total time per trial that an individual spent inside the feeding arena.
- Co-feeding: Two or more individuals eating from the same piece of banana at the same time. Note that this definition of co-feeding differs from the one used for chimpanzees^4^, bonobos^3^ and lemurs^7^, which defined it as two or more individuals eating at the same time on the arena. This definition could not be used for the marmosets because it resulted in ceiling effects.
- Food sharing: A change of food possession from one individual to the other.
- Moving to the edge: Any time an individual removed a piece of food from its original position and carried it to the edge of the arena.
- Leaving with food: Any time an individual removed a piece of food from its original position and left the arena with it.
- Leaving without food: Any time an individual left the arena without food.
-Non-contact aggression: Aggressive behaviour without physical contact, either in the form of chasing or vocalizations (chatter calls, can be accompanied with upright body position).
- Contact aggression: Agonistic behaviours with physical contact (pushing away, biting).

We then quantified for each non-infant individual the ***time spent inside the feeding arena***, and the frequencies of ***food tolerance*** (i.e. the sum of co-feeding and food sharing), ***evasive behaviours*** (the sum of moving banana pieces to the arena’s edge, leaving the arena with a piece of banana, and leaving the arena without a piece of banana), and ***agonistic behaviours*** (threat behaviours and aggression).

We first performed a Principal Component Analysis (PCA) using the behavioural categories defined above. We used the nFactors package to determine the number of components to be extracted (Supplementary Figure 1). This parallel analysis suggested the extraction of two factors. The first factor was named as ***spatial tolerance¸*** with high positive loading of *time spent on the arena* and negative loadings of *evasive behaviours*, the second factor corresponded to ***feeding tolerance,*** with positive loadings of *co-feeding and food sharing behaviours*. We used these two non-rotated PCA factors, dubbed “spatial tolerance” and “feeding tolerance” for subsequent Linear Mixed Model analyses, using PC scores as our dependent variable.

For all models, we use *environment* (wild/captivity), *sex-status* (female breeder/male breeder/female helper/male helper), *presence of infants in the group* (yes/no), and *sex ratio* (number of adult males: adult females) as fixed effects. For models on PC 1 (***spatial tolerance)*** we use individuals nested in group as random effects and for models on PC 2 (***feeding tolerance)*** we only use group as random effect. Specifically, to investigate other possible confounding effects, our models also included infant presence and sex ratio as fixed factors, because although these variables were not the aim of this study, both could have an effect on social tolerance. We compared all possible models and report the ones with the lowest AIC value (Table 2 and Supplementary Tables 3, 5, 7 and 9) after performing the *step* function. All statistical analyses were performed in R version 3.5.2. We also performed a Games-Howell post-hoc test for each PCA factor within conditions for every pair-wise comparison on Sex-Status categories for ***spatial tolerance*** and every Status category for ***feeding tolerance*** (Supplementary Tables 1 and 2).

Finally, to corroborate the results from analysing ***spatial tolerance*** and ***feeding tolerance***, we performed the same model selection approach, described above, for the separate behaviours (Supplementary Tables 3–10 and Supplementary Figures 2–5). For all behavioural models we also used trial duration as an offset term. Time spent in the arena and the offset term were both log transformed on model 1.

## Experiment statement

All animals were kept in accordance with Swiss legislation. All experiments were in concordance with the ethical regulations both in Brazil from Federal University of Rio Grande do Norte ethics committee (#067.085/2017) and Switzerland from the Kantonales Veterinäramt (#183/13). Fieldwork was carried out in accordance to brazilian fieldwork permission from Instituto Chico Mendes de Conservação da Biodiversidade (#59,828).

## Methodological statement

All methods in this manuscript were carried out in accordance with relevant guidelines and regulations cited in the experiment statement above.

## Supplementary Information

Supplementary Information.

## Data Availability

The data that support the findings of this study are available from the corresponding author upon reasonable request.
