# Supplementary material for: Higher social tolerance in wild versus captive common marmosets: the role of interdependence
Source: Sci Rep. 2021 Jan 12;11:825. doi: 10.1038/s41598-020-80632-3 (PMC7804027; doi:10.1038/s41598-020-80632-3)
Supplement: Supplementary file 1 — Supplementary Information. [file 41598_2020_80632_MOESM1_ESM.docx]

**Supplementary Material**

**Title:**

**Higher social tolerance in wild versus captive common marmosets: the role of interdependence**

**Author line:** de Oliveira-Terceiro, Francisco Edvaldo*^1,2^; Arruda, Maria de Fátima^1^; van Schaik, Carel P.^2^; Araújo, Arrilton^1^; Burkart, Judith Maria^2^

**Author affiliation:** 1: Department of Physiology and Behaviour, Universidade Federal do Rio Grande do Norte, Av. Sen. Salgado Filho, 3000 - Candelária, Natal - RN, 59064-741, Brazil

2: Department of Anthropology, Universität Zürich, Winterthurerstrasse 190, 8057 Zürich, Switzerland

**Corresponding author:** de Oliveira-Terceiro, Francisco Edvaldo; Av. Sen. Salgado Filho, 3000 - Candelária, Natal - RN, 59064-741, Brazil; +55(84) 99421-8008; [deoliveiraterceiro@gmail.com](mailto:deoliveiraterceiro@gmail.com) ORCID: https://orcid.org/0000-0002-9071-604X

**A) Parallel analysis for PCA of separate variables**


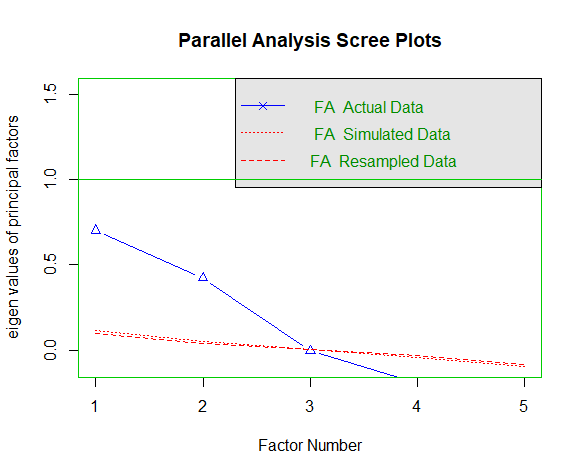
 **Figure 1:** Parallel analysis to determine number of dimensions from Principal Component Analysis. Software R Studio version 1.1.463. https://www.r-project.org/

**B) Post-hoc analyses for *Spatial tolerance***

**Table 1:** Games-Howell post hoc test for spatial tolerance (PC 1)

| ***spatial tolerance*** | Games-Howell post hoc test | | |
| --- | --- | --- | --- |
| Pair-wise comparison | Mean difference | t Value | p Adjusted Value |
| Wild female breeder x Captive female breeder | -0.17 | 1.06 | 1 |
| Wild male breeder x Captive male breeder | -0.58 | 4.47 | **0.014*** |
| Wild female helper x Captive female helper | -0.94 | 6.18 | **0.0001***** |
| Wild male helper x Captive male helper | -0.93 | 5.53 | **0.0001***** |

**C) Post-hoc analyses for *Feeding tolerance***

**Table 2:** Games-Howell post hoc test for feeding tolerance (PC 2)

| ***feeding tolerance*** | Games-Howell post hoc test | | |
| --- | --- | --- | --- |
| Pair-wise comparison | Mean difference | t Value | p Adjusted Value |
| Wild breeders x Captive breeders | 0.42 | 6.33 | **0.001**** |
| Wild helpers x Captive helpers | 0.89 | 8.23 | **0.001**** |

**D) Behavioural analyses**

**D1) Time Spent on arena**

**Time spent on arena:** To estimate each group tolerance we first analysed time spent inside the feeding arena by each individual as dependent variable. We used a Linear Mixed-effects model (LME), having individual nested in group as random effect and total trial duration as an offset term after log transforming time measures (model 1 in Table 3). Marmosets housed in captivity spent less time on the feeding arena than their wild counterparts (Figure 2). Furthermore, this difference is stronger when comparing Sex-Status categories across environments (X² (9) =24.346, p < 0.001).

**Table 3:** Anova table of model on time spent on arena. Bold values indicate p < 0.05

| **Fixed factor** | NumDF | DenDF | F-value | p-value |
| --- | --- | --- | --- | --- |
| **Model 1: Time spent on arena** | | | | |
| *Sex-Status* | 3 | 30342 | 7.224 | **0.0009 ***** |
| *Environment* | 1 | 5389 | 6.361 | **0.05 *** |
| Infant presence | 1 | 26477 | 0.027 | 0.870 |
| Sex ratio | 1 | 33813 | 1.217 | 0.278 |
| *Environment*Sex-Status* | 3 | 30345 | 3.583 | **0.025 *** |

**Table 4:** Post hoc Games-Howell test on Time inside arena comparing each Sex-Status category within condition (FB – Female Breeder; MB – Male Breeder; FH – Female Helper; MH – Male Helper). Bold values indicate p < 0.05

| Time spent on arena | Wild condition | | | Captive condition | | |
| --- | --- | --- | --- | --- | --- | --- |
| Pair-wise comparison | Mean difference | t Value | p Adjusted Value | Mean difference | t Value | p Adjusted Value |
| MB-FB | 1.83 | 0.30 | 1 | -21.80 | 3.74 | **.010 **** |
| FH-FB | -4.11 | 0.89 | 1 | -26.13 | 5.02 | **.0001 ***** |
| MH-FB | -5.69 | 1.18 | 1 | -34.37 | 6.73 | **.0001 ***** |
| MH-MB | -5.94 | 1.14 | 1 | -4.33 | 1.12 | 1 |
| FH-MB | -7.51 | 1.39 | 1 | -12.56 | 3.37 | **.035 *** |
| MH-FH | -1.57 | 0.45 | 1 | -8.24 | 3.11 | .067 |


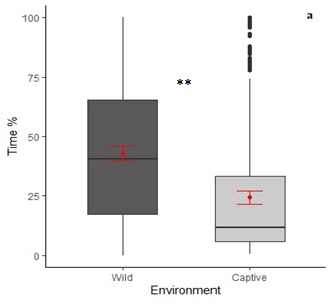


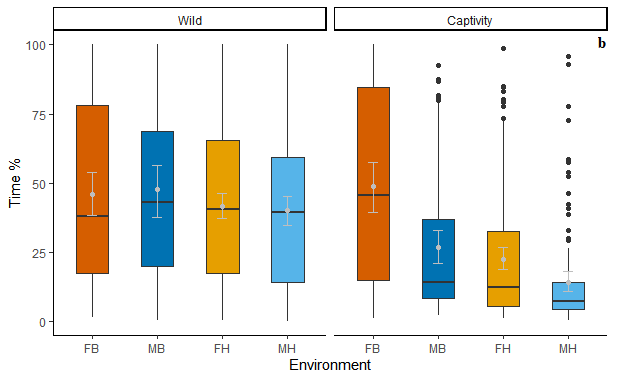


**Figure 2: Time spent on arena**, split up according to a) environment; black lines indicate medians; upper and lower boxes indicate upper and lower quartiles; whiskers indicate the ranges for the bottom and top 25% of the data values, excluding outliers; black dots indicate outliers; red dot indicate mean value and red bars indicate SEM; b) sex-status categories in the wild and captivity. Black lines indicate medians; upper and lower edges of boxes indicate upper and lower quartiles; whiskers indicate maximum and minimum values; black dots indicate outliers; gray dots indicate mean values, and gray bars indicate SEM. *: p <0.05, **: p<0.01, ***: p<0.001 (Games-Howell post-hoc tests; FB – Female Breeder; MB – Male Breeder; FH – Female Helper; MH – Male Helper). Software R Studio version 1.1.463. https://www.r-project.org/

**D2) Evasive behaviour**

**Evasive behaviour:** We used a Generalized Linear Mixed-effects model (GLMM) having individual nested in group as random effect and total trial duration as an offset term for evasive behaviour. Wild-living animals were more tolerant, evading less frequently than captive marmosets (X² (7) =55.991, p < 0.001; Figure 3 and model 2 in Table 5). Although every Sex-Status category had higher evasion rates in captive conditions than in the wild, the rate across Sex-Status retain similar pattern across environments, the only difference being between breeders and helpers (Figure 3 and Table 6). Infant presence had different impacts across environment, most notably the lower incidence of evasive behaviour in captivity from groups with infants if compared to wild-living groups (model 2 in Table 5 and Figure 3).

**Table 5:** Anova table of model on Evasive behaviours. Bold values indicate p < 0.05

| **Fixed factor** | DF | Chisq | p-value |
| --- | --- | --- | --- |
| **Model 2: Evasive behaviours** | | | |
| *Sex-Status* | 3 | 20.189 | **0.0001 ***** |
| *Environment* | 1 | 84.799 | **< 2.2e-16 ***** |
| *Infant presence* | 1 | 31.570 | **1.924e-08 ***** |
| *Sex ratio* | 1 | 63.454 | **1.64e-15 ***** |
| *Environment*Infant presence* | 1 | 23.278 | **1.402e-06 ***** |

**Table 6:** Post hoc Games-Howell test on Evasive behavior comparing each Sex-Status category within condition - (FB – Female Breeder; MB – Male Breeder; FH – Female Helper; MH – Male Helper). Bold values indicate p < 0.05

| Evasive behaviours | Wild condition | | | Captive condition | | |
| --- | --- | --- | --- | --- | --- | --- |
| Pair-wise comparison | Mean difference | t Value | p Adjusted Value | Mean difference | t Value | p Adjusted Value |
| MB-FB | 0.00 | 1.10 | 1 | -0.01 | 1.07 | 1 |
| FH-FB | 0.00 | 2.34 | .538 | 0.01 | 0.80 | 1 |
| MH-FB | 0.01 | 2.37 | .510 | 0.05 | 2.50 | .371 |
| MH-MB | 0.00 | 0.80 | 1 | 0.03 | 2.69 | .218 |
| FH-MB | 0.01 | 1.58 | 1 | 0.06 | 4.04 | **.002 **** |
| MH-FH | 0.01 | 1.11 | 1 | 0.03 | 2.24 | .678 |


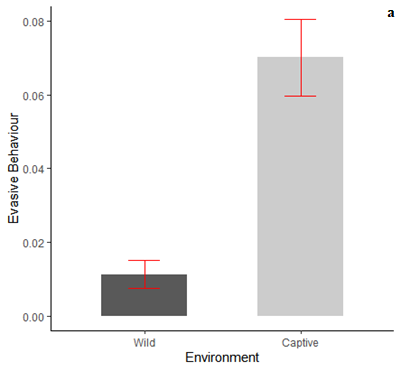


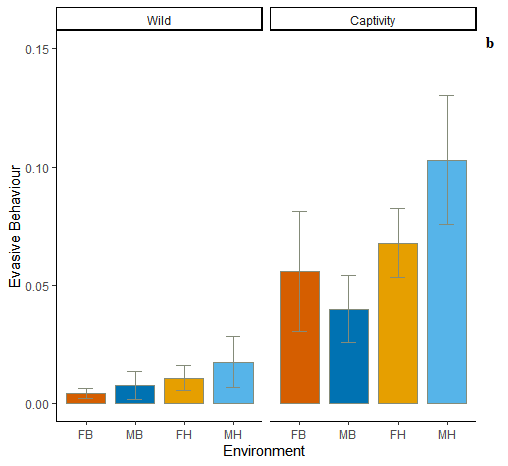


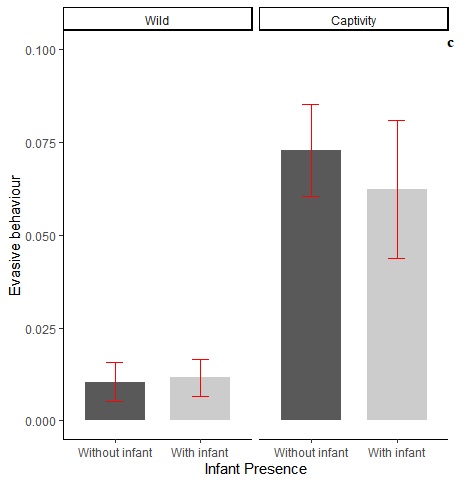


**Figure 3: Evasive behaviour**, split up according to a) environment, b) sex-status categories in the wild and captivity, and c) infant presence in each environment. Error bars are mean ± SEM on both colours. *: p <0.05, **: p<0.01, ***: p<0.001 (Games-Howell post-hoc tests; FB – Female Breeder; MB – Male Breeder; FH – Female Helper; MH – Male Helper). Software R Studio version 1.1.463. https://www.r-project.org/

**D3) Food sharing and Co-feeding**

**Food sharing and Co-feeding:** We measured Food tolerance, both co-feeding and food sharing as explained in the methods section. We used a Generalized Linear Mixed-effects model (GLMM) having individual nested in group as random effect and total trial duration as an offset term, as pointed in models 3a and 3b on Table 7. Following the pattern found in arena permanence, co-feeding was also greater in groups in the natural environment (GLMM, (X² (8) =31.092, p < 8.984e-06; Figure 4). However, our model on food sharing showed no difference to the null model for any of the factors. (model 3 in Table 7; See also Table 8 and figure 4).

**Table 7:** Anova table of models on Food sharing and co-feeding. Bold values indicate p < 0.05

| **Fixed factor** | DF | Chisq | p-value |
| --- | --- | --- | --- |
| **Model 3a: Food sharing** | | | |
| Sex-Status | 3 | 0.233 | 0.972 |
| Environment | 1 | 0.085 | 0.771 |
| Infant presence | 1 | 0.888 | 0.346 |
| Sex ratio | 1 | 0.691 | 0.406 |
| **Model 3b: Co-feeding** | | | |
| Sex-Status | 3 | 7.720 | 0.052 |
| *Environment* | 1 | 15.038 | **0.0001***** |
| *Sex ratio* | 1 | 14.108 | **0.0001***** |

**Table 8:** Post hoc Games-Howell test on Food tolerance comparing each Sex-Status category within condition - (FB – Female Breeder; MB – Male Breeder; FH – Female Helper; MH – Male Helper).

| Food sharing | Wild condition | | | Captive condition | | | |
| --- | --- | --- | --- | --- | --- | --- | --- |
| Pair-wise comparison | Mean difference | t Value | p Adjusted Value | Mean difference | t Value | | p Adjusted Value |
| MB-FB | 0.00 | 0.28 | 1 | 0.00 | 1.36 | | 1 |
| FH-FB | 0.00 | 1.80 | 1 | 0.00 | 2.29 | | .611 |
| MH-FB | 0.01 | 1.42 | 1 | 0.00 | 1.80 | | 1 |
| MH-MB | 0.00 | 1.73 | 1 | 0.00 | 2.08 | | .971 |
| FH-MB | 0.00 | 1.38 | 1 | 0.00 | 1.45 | | 1 |
| MH-FH | 0.00 | 0.45 | 1 | 0.00 | 1.00 | | 1 |
| Co-feeding | Wild condition | | | Captive condition | | | |
| Pair-wise comparison | Mean difference | t Value | p Adjusted Value | Mean difference | | t Value | p Adjusted Value |
| MB-FB | 0.00 | 2.64 | .289 | 0.00 | | 0.00 | 1 |
| FH-FB | 0.01 | 2.10 | .937 | 0.00 | | 0.00 | 1 |
| MH-FB | 0.01 | 1.83 | 1 | 0.00 | | 0.00 | 1 |
| MH-MB | 0.00 | 0.51 | 1 | 0.00 | | 0.00 | 1 |
| FH-MB | 0.00 | 0.48 | 1 | 0.00 | | 0.00 | 1 |
| MH-FH | 0.00 | 0.02 | 1 | 0.00 | | 0.00 | 1 |


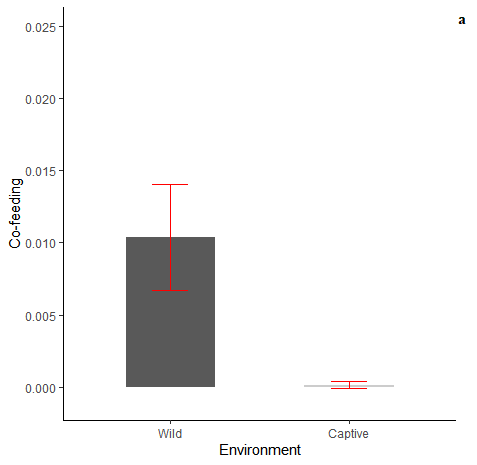


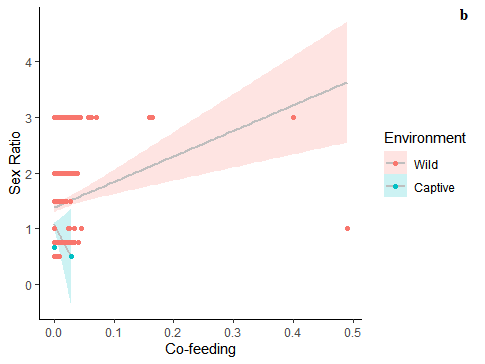


**Figure 4: Co-feeding**, split up according to a) environment; error bars are mean ± SEM; b) sex ratio and Environment; red dots indicate Co-feeding occurrences in wild condition blue dots indicate co-feeding occurrences in the captive condition *: p <0.05, **: p<0.01, ***: p<0.001. Software R Studio version 1.1.463. https://www.r-project.org/

**D4) Agonism**

**Agonism:** We did not find a significant difference for agonistic behaviour due to environment condition. (Figure 5). Our model was significantly better than our null model due to Sex-Status differences (model 4 in Table 9). Both male breeders and helpers engaged in more agonistic events than their female counterparts (X² (4) =9.9.313, p < 0.0416; Figure 5 and Table 9 and 10). Our model based on agonistic behaviour, unlike the other models in this study, does not consider sex-ratio and infant presence due to statistical limitations derived from lack of data variance on these categories.

**Table 9:** Anova table of model on Agonism. Bold values indicate p < 0.05

| **Fixed factor** | DF | Chisq | p-value |
| --- | --- | --- | --- |
| **Model 4: Agonism** | | | |
| *Sex-Status* | 3 | 12.815 | **0.005 **** |
| Environment | 1 | 1.794 | 0.180395 |

**Table 10:** Post hoc Games-Howell test on Agonistic behavior comparing each Sex-Status category within condition - (FB – Female Breeder; MB – Male Breeder; FH – Female Helper; MH – Male Helper).

| Agonism | Wild condition | | | Captive condition | | |
| --- | --- | --- | --- | --- | --- | --- |
| Pair-wise comparison | Mean difference | t Value | p Adjusted Value | Mean difference | t Value | p Adjusted Value |
| MB-FB | 0.00 | 2.88 | .131 | 0.00 | 1 | 1 |
| FH-FB | 0.00 | 0.25 | 1 | 0.00 | 2.11 | .914 |
| MH-FB | 0.00 | 2.49 | .377 | 0.00 | 1.64 | 1 |
| MH-MB | 0.00 | 2.42 | .449 | 0.00 | 2.07 | .979 |
| FH-MB | 0.00 | 0.63 | 1 | 0.00 | 1.59 | 1 |
| MH-FH | 0.00 | 2.03 | 1 | 0.00 | 0.88 | 1 |


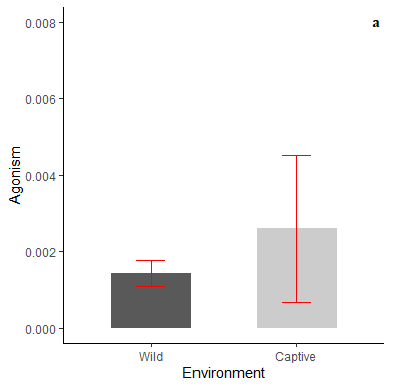


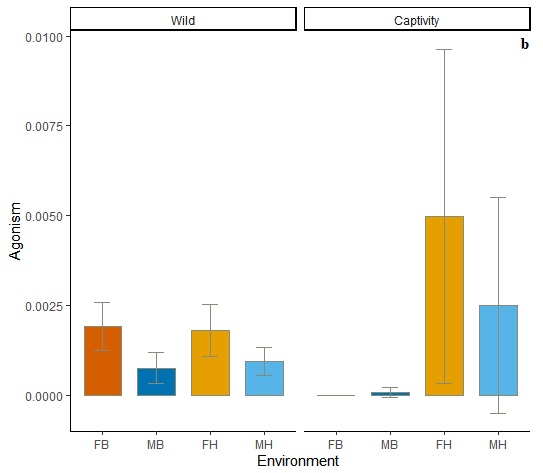


**Figure 5: Agonism**, split up according to a) environment, b) sex-status categories in wild condition and in captive condition. Error bars are mean ± SEM *: p <0.05, **: p<0.01, ***: p<0.001 (Games-Howell post-hoc tests; FB – Female Breeder; MB – Male Breeder; FH – Female Helper; MH – Male Helper). Software R Studio version 1.1.463. https://www.r-project.org/

**E) Influence of participation**

**Experiment participation:** First, we conducted a binomial GLMM to assess any effects on experiment participation (in Table 12, model 5). Neither environment nor Sex-Status category influenced the marmosets’ participation in the experiment (Figure 6 and model 5 in Table 12), whereas Sex Ratio and Infant presence positively affected experiment participation in the captive condition (X² (6) =15.234, p=0.0185 – model 5 in Table 12).

**Table 11:** Group composition and experiment participation rate

| **Environment** | **Group** | **Individual** | **Sex-Status** | **Participation (%)** |
| --- | --- | --- | --- | --- |
| Captivity | Jaja | Jaja | Female Breeder | 85 |
|  |  | Membo | Male Breeder | 95 |
|  |  | Jandira | Female Helper | 90 |
|  |  | Jala | Male Helper | 100 |
|  |  | Jelly | Female helper | 100 |
|  | Mina | Mina | Female Breeder | 95 |
|  |  | John | Male Breeder | 100 |
|  |  | Merkur | Male Helper | 100 |
|  |  | Mojita | Female Helper | 100 |
|  | Nikita | Nikita | Female Breeder | 95 |
|  |  | Puk | Male Breeder | 90 |
|  |  | Ninja | Male Helper | 95 |
|  |  | Nunchaku | Male Helper | 100 |
|  |  | Nashi | Female Helper | 60 |
|  |  | Nori | Male Helper | 95 |
|  |  | Nyoko | Infant Female | - |
|  |  | Natsuki | Infant Female | - |
|  | Lex | Lex | Male Helper | 50 |
|  |  | Nebula | Female Helper | 45 |
|  |  | Nutela | Female Helper | 95 |
|  |  | Nougat | Male Helper | 90 |
|  |  | Nox | Female Helper | 90 |
|  |  | Nux | Female Helper | 100 |
| Wild | Torre | Tavira | Female Breeder | 91.67 |
|  |  | Tiago | Male Breeder | 95.83 |
|  |  | Tómas* | Male Helper | 33.33 |
|  |  | Tejo | Male Helper | 87.5 |
|  |  | Taís | Female Helper | 100 |
|  |  | Tônia | Male Helper | 100 |
|  |  | Tobias | Infant Male | - |
|  |  | Teresa | Infant Female | - |
|  | Bosque | Catarina | Female Breeder | 72.41 |
|  |  | Baggi | Female Breeder | 68.97 |
|  |  | Bolton | Male Breeder | 89.66 |
|  |  | Bruce | Male Helper | 65.52 |
|  |  | Barriston | Male Helper | 65.52 |
|  |  | Bali** | Female Helper | 51.72 |
|  |  | Bahia | Female Helper | 65.52 |
|  |  | Belinda** | Female Helper | 17.24 |
|  |  | Botafogo | Male Helper | 82.76 |
|  |  | Buarque | Male Helper | 58.62 |
|  |  | Beta | Female Helper | 89.66 |
|  |  | Brasilia | Female Helper | 82.76 |
|  |  | Biana* | Infant Female | 55.17 |
|  |  | Brando* | Infant Male | 13.79 |
|  |  | Benfica* | Infant Male | 13.79 |
|  |  | Bia | Infant Female | - |
|  |  | Beja | Infant Female | - |

*Individuals that became helpers during experiments. ** Individuals that left the group or died during experiments

**Table 12:** Anova table of model on Experiment participation. Bold values indicate p < 0.05

| **Fixed factor** | DF | Chisq | p-value |
| --- | --- | --- | --- |
| **Model 5: Experiment participation** | | | |
| Environment | 1 | 20.189 | 0.994 |
| Sex-Status | 3 | 1.161 | 0.762363 |
| *Infant presence* | 1 | 4.912 | **0.027 *** |
| *Sex ratio* | 1 | 10.006 | **0.002 **** |


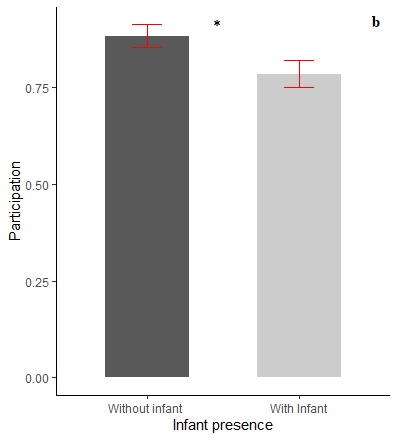

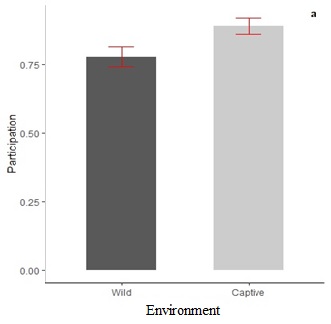


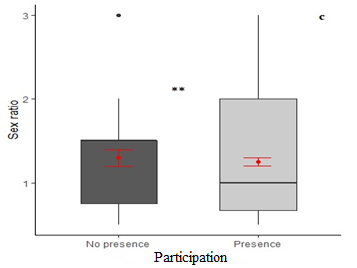


**Figure 6: Experiment participation**, split up according to a) environment, b) infant presence; error bars are mean ± SEM, and c) sex ratio. Black lines indicate medians; upper and lower edges of boxes indicate upper and lower quartiles; whiskers indicate the ranges for the bottom and top 25% of the data values, excluding outliers; black dots indicate outliers; red dots indicate mean value and red bars indicate SEM. *: p <0.05, **: p<0.01, ***: p<0.001. Software R Studio version 1.1.463. https://www.r-project.org/


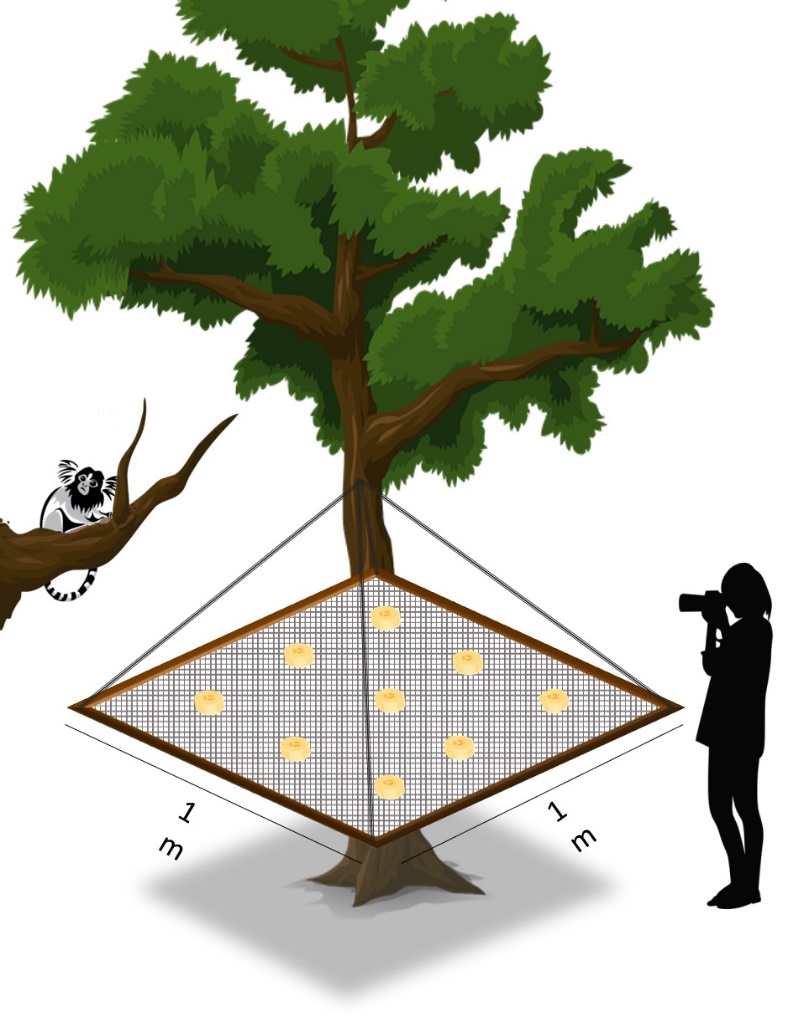


**Figure 7: Experiment apparatus,** representation of one-square meter experiment arena and researcher video recording experiments. Each session started when the first marmoset entered the arena and it would be considered finished when no piece of banana was left in the arena. Software Power Point version 2011. https://cutt.ly/yhJsdPe
